# The Impact of Structured and Standardized Documentation on Documentation Quality; a Multicenter, Retrospective Study

**DOI:** 10.1007/s10916-022-01837-9

**Published:** 2022-05-27

**Authors:** Tom Ebbers, Rudolf B. Kool, Ludi E. Smeele, Richard Dirven, Chrisje A. den Besten, Luc H. E. Karssemakers, Tim Verhoeven, Jasmijn M. Herruer, Guido B. van den Broek, Robert P. Takes

**Affiliations:** 1 Department of Otorhinolaryngology and Head and Neck Surgery, Radboud University Medical Center, Nijmegen, Netherlands; 2 Department of Head and Neck Oncology and Surgery, Antoni Van Leeuwenhoek, Amsterdam, Netherlands; 3 Radboud University Medical Center, Radboud Institute for Health Sciences, IQ Healthcare, Nijmegen, Netherlands; 4 Department of Oromaxillofacial Surgery and Head and Neck Surgery, Radboud University Medical Center, Nijmegen, Netherlands

**Keywords:** Electronic health record, Structured and standardized documentation, Structured documentation, Documentation quality, Data reuse

## Abstract

The reuse of healthcare data for various purposes will become increasingly important in the future. To enable the reuse of clinical data, structured and standardized documentation is conditional. However, the primary purpose of clinical documentation is to support high-quality patient care. Therefore, this study investigated the effect of increased structured and standardized documentation on the quality of notes in the Electronic Health Record. A multicenter, retrospective design was used to assess the difference in note quality between 144 unstructured and 144 structured notes. Independent reviewers measured note quality by scoring the notes with the Qnote instrument. This instrument rates all note elements independently using and results in a grand mean score on a 0–100 scale. The mean quality score for unstructured notes was 64.35 (95% CI 61.30–67.35). Structured and standardized documentation improved the Qnote quality score to 77.2 (95% CI 74.18–80.21), a 12.8 point difference (p < 0.001). Furthermore, results showed that structured notes were significantly longer than unstructured notes. Nevertheless, structured notes were more clear and concise. Structured documentation led to a significant increase in note quality. Moreover, considering the benefits of structured data recording in terms of data reuse, implementing structured and standardized documentation into the EHR is recommended.

**Supplementary information:**

The online version contains supplementary material available at 10.1007/s10916-022-01837-9.

## Introduction

Clinical documentation is the process of creating a text record that summarizes the interaction between patients and healthcare providers during clinical encounters [1]. The quality of clinical documentation is important as it impacts quality of patient care, patient safety, and the number of medical errors [2–4]. Furthermore, clinical documentation is increasingly used for other purposes, such as quality measurement, finance, and research. Additionally, regulatory requirements regarding documentation have increased [5, 6]. Consequently, physicians are spending more and more time on documentation [7].

In recent years, various tools and techniques have been developed to increase documentation efficiency and decrease the time physicians need to spend on documentation. These techniques are known as content importing technology (CIT). Examples of CIT are copy and paste functions (CPF), automated data import from other parts of the electronic health record (EHR), templates, or macros. These tools seem to have multiple benefits, primarily faster documentation during patient visits. However, Weis and Levy described that the use of CIT has multiple risks. Incorrect insertion of data from other parts of the record, or excessively long, bloated notes can distract a reader from key, essential facts and data [8]. However, when used correctly, it should be possible to limit these risks.

In addition to the need to increase documentation efficiency, documentation needs to be accurate. Cohen et al. stated that variation in EHR documentation between physicians impedes effective and safe use of EHRs, emphasizing the need for increased standardization of documentation [9]. However, some studies have suggested that structured and standardized documentation *(hereafter: structured documentation)* can impede expressivity in notes. Rosenbloom explored this tension between flexible, narrative documentation and structured documentation and recommended that healthcare providers can choose how to document patient care based on workflow and note content needs [1]. This implies that structured documentation is preferred when reuse of data is desirable. On the other hand, narrative documentation can be used when reuse of information is not required.

Research has shown that structured documentation can improve provider efficiency and decrease documentation time [10]. Unfortunately, little is known about the effects that a transition from primarily unstructured, free-text EHR documentation to structured and standardized EHR documentation has on the quality of EHR notes. To date, research on this topic has mainly focused on the difference between paper-based and electronic documentation [11–13]. Although reuse of data, for which structured documentation is essential, will become increasingly important, the primary goal of EHR documentation is supporting high-quality patient care [14]. Therefore, the primary objective was to investigate the effect of increased standardized and structured documentation on the quality of EHR notes.

## Methods

Since 2009, the Radboudumc Center for Head and Neck Oncology developed and implemented a highly structured care pathway. A care pathway is a complex intervention for the mutual decision-making and organization of care processes for a well-defined group of patients during a well-defined period [15]. In 2017, for all stages of the care pathway (e.g. first visit consultation, multidisciplinary tumor board, diagnostic results consultation, treatment, follow-up consultation) the patient information that had to be entered into the EHR was defined. Structured and standardized forms using different types of CIT, automated documentation and standardized response options were developed in Epic EHR (EPIC, Verona Wisconsin). These forms allowed physicians to enter all patient information efficiently into the EHR. This resulted in structured and standardized notes while simultaneously storing structured data elements into the EHR database. These data elements can be reused in other stages of the care pathway, automatically compute referral letters, trigger standardized ordersets, or other tools to make the care process more efficient. Ultimately, this data is used to populate real-time quality dashboards. Furthermore, data can be extracted from the EHR and sent to third parties, such as quality and cancer registries or other health care centers when referring patients. Besides structured data recording, these forms support additional narrative documentation if needed or preferred. Recently, a similar highly structured care pathway with structured documentation based on the previously developed care pathway in Radboudumc, was implemented at the Head and Neck Oncology department in Antoni van Leeuwenhoek. In this center, HiX EHR (Chipsoft, Amsterdam) is used. Because of the difference in EHR vendor and the resulting variation in technical possibilities of the EHRs, there were slight differences in structured forms and notes in both centers. However, the structured forms that were built in center B remain highly similar to the forms used in Center A, as the forms and notes of Center A were shared with center B and were subsequently used in the development phase.

A multicenter, retrospective design was used to assess the difference in note quality in two tertiary HNC care centers. In center A, structured documentation has gradually increased in recent years. Therefore, the EHR notes of patients seen between January and December 2013 were compared with those of patients seen between January and December 2019. The transition to structured documentation in center B was more immediate due to implementing an EHR embedded care path that supports structured documentation. Therefore, the notes of patients seen between March and July 2020 were compared with those seen between January and April 2021. This shorter interval added to internal validity because it is less likely that other, time-related factors influenced the outcome. Notes of consultations of adult patients that completed at least one initial oncological consultation (IOC) or follow-up consultation (FUC) during the study period were eligible for inclusion. In both centers, a list of eligible notes was extracted from the EHR and for each consultation type and each documentation method, 36 notes were randomly drawn. In total, 288 notes were included. Subsequently, notes were carefully anonymized. All names, dates, and other identifying information were replaced with < name > , < date > , or otherwise masked. A translated example of a structured note is available as Electronic Supplementary Material (Online Resource 1). HNC care providers from center A were recruited to rate the notes collected in center B, and HNC care providers in center B were recruited to rate notes from center A to minimize bias. Each physician was assigned a random group of notes. However, unstructured and structured notes were evenly distributed among raters. Subsequently, notes were scored in a secured digital environment created in CastorEDC (Castor, Amsterdam), an electronic data capture platform.

The quality of the notes was assessed using the Qnote instrument, a validated measurement method for the quality of clinical documentation [16]. This instrument rates every element of a note individually, by using one or more of seven components (Table 1).

**Table 1:** Elements and components of Qnote instrument

**Elements**	**Components**
Chief complaint	Sufficient information
History of present illness	Concise
Problem list	Clear
Past medical history	Organized
Medications	Complete
Adverse drug reactions and allergies	Ordered
Social and family history	Current
Review of systems	
Physical findings	
Assessment	
Plan of care	
Follow-up information	

The primary outcomes of this study were the quality of notes and note elements, measured by the Qnote instrument on a 100-point scale. Secondary outcomes included length of notes in words, mean component scores per note, and subjective quality measured by a general score given on a scale of 1–10.

Data were notated and analyzed using SPSS version 25 (IBM Corp, Armonk, NY, USA). Two-way ANOVA was used to assess differences in note quality between before and after implementation of structured documentation. The Qnote grand mean score and element scores were outcome variables. The type of note, the originating center, and a dummy variable indicating the period in which the note was written were added as fixed factors. Two-tailed significance was defined as p < 0.05 or a 95% CI not including zero.

This study was approved by the Institutional Review Boards at Antoni van Leeuwenhoek Netherlands Cancer Institute and Radboud University Medical Center.

## Results

The grand mean score of all 144 EHR notes written before implementing structured documentation was 64.35 (95% CI 61.30–67.35). When comparing this score to all 144 EHR notes written with structured documentation, a 12.8 point difference (p < 0.001) was found. Structured documentation improved the grand mean score to 77.2 (95% CI 74.18–80.21). Subsequently, additional analysis was conducted on all element scores. The results are shown in Table 2.

**Table 2:** Estimated marginal means of Qnote scores and main effect of structured documentation

**Element**	**Qnote score** **Unstructured documentation**	**Qnote score** **Structured documentation**	**Mean difference (95% CI)**	**p-value of difference**
Chief complaints	84.0	93.3	+9.3 (4.0 to 14.7)	0.001*
HPI	71.6	87.1	+15.4 (7.8 to 23.1)	0.000*
Problem list	23.3	39.0	+15.7 (3.9 to 27.6)	0.009*
Past medical history	38.8	47.0	+8.2 (0.0 to 16.4)	0.050*
Medications	29.5	42.0	+12.6 (–3.3 to 28.4)	0.120
Adverse reactions	25.6	84.7	+59.1 (47.2 to 71.0)	0.000*
Social and family history	72.5	88.3	+15.8 (6.3 to 25.5)	0.001*
Physicial findings	82.8	85.3	+2.5 (–2.2 to 7.2)	0.293
Assessment	74.5	85.9	+11.4 (5.1 to 17.7)	0.000*
Plan of Care	74.5	80.1	+5.7 (–2.3 to 13.7)	0.162
Follow-up information	72.5	86.9	+14.4 (7.9 to 20.9)	0.000*
**Grand Mean**	**64.4**	**77.2**	**+12.8 (8.7–17.0)**	0.000*

* difference significant (p < 0.05)

**Fig. 1:**
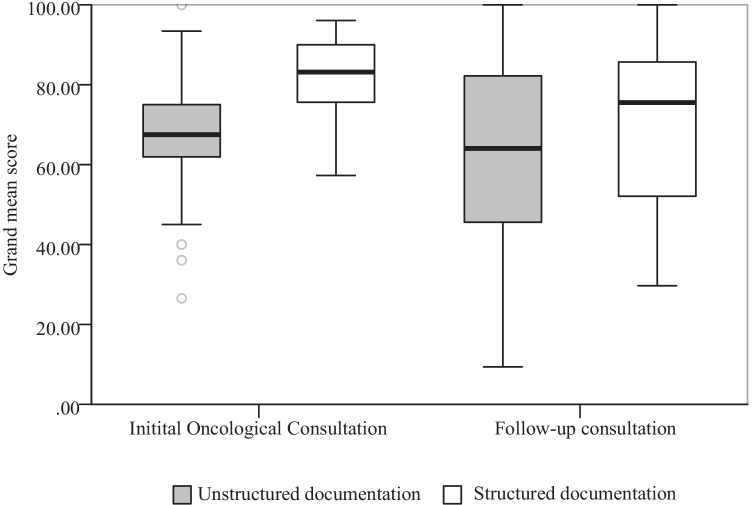
Boxplot of grand mean score per note type

Table 3 shows descriptive results of element scores displayed per type of note. What can be observed from the data in Table 3 is that for structured documentation, the standard deviation decreases in most elements scores, indicating the variability in quality seems to be lower in structured notes. Furthermore, when comparing the grand mean score for IOC and FUC notes separately, an increase for both types of notes was found (Fig. 1). IOC Qnote score increased by 14.9 (95% CI 11.3–18.5) points from 67.3 to 82.3. FUC Qnote score increased by 10.8 (95% CI 4.6–17.0) from 61.3 to 72.1.

**Table 3:** Descriptive results of Qnote element scores, per note type

	**Initital Oncological Consultation**				**Follow-up consultation**			
	**Unstructured**		**Structured**		**Unstructured**		**Structured**	
	**Mean**	**(SD)**	**Mean**	**(SD)**	**Mean**	**(SD)**	**Mean**	**(SD)**
Chief complaints	89,4	(22,2)	97,2	(11,5)	78,6	(30,2)	89,4	(23,8)
HPI	87,4	(27,7)	97,4	(8,6)	55,8	(46,4)	76,7	(36,3)
Problem list	33,8	(46,6)	46,5	(49,0)	12,7	(33,1)	31,5	(45,8)
Past medical history	73,7	(41,5)	85,2	(31,6)	4,7	(19,1)	8,0	(26,6)
Medications	29,5	(45,3)	42,0	(49,5)	*			
Adverse reactions	25,6	(40,0)	84,7	(31,1)	*			
Social and family history	72,5	(36,2)	88,3	(19,4)	*			
Physicial findings	87,3	(15,5)	87,0	(16,4)	78,2	(26,5)	83,6	(20,6)
Assessment	83,3	(20,6)	88,3	(18,7)	65,8	(39,3)	83,6	(23,5)
Plan of Care	80,1	(25,1)	89,6	(17,3)	69,3	(41,0)	69,9	(43,4)
Follow-up information	63,9	(32,1)	88,0	(22,0)	81,0	(27,9)	85,7	(27,1)
Grand Mean	67,4	(12,6)	82,3	(8,7)	61,3	(25,4)	72,1	(20,2)

* grey marked elements were not evaluated for this note because these elements were considered not relevant in this type of consultation

Subsequently, analysis was conducted on data from both centers separately to determine whether structured documentation led to increased quality in both centers. In center B, an increase of 14.59 was found (95% CI 7.22–21.96) in IOC note quality, and a 16.36 point increase (95% CI 8.99–23.73) in FUC note quality was found. A significant improvement in IOC Qnote score by 15.10 (95% CI 8.26–22.10) was observed in center A. The 5.3 point increase in FUC note quality was not statistically significant (95% CI -1.61–12.14).

Analysis of secondary outcome measures showed a significant increase in note length for structured documentation in both note types. IOC notes increased from 442.1 to 639.6 words, with a mean difference of 197.5 (95% CI 146.9–248.1), translating to a 44.7% increase. A significant 53.3% increase was found in FUC notes, increasing with 46.5 words (95% CI 31.7–61.2) from 86.9 to 133.4. To evaluate whether this increase in note length led to unnecessary long notes containing excessive non-essential information, all scores for a given component were averaged. For example, the component concise was used to rate 9 of the 11 elements used to rate a note. The mean of all conciseness scores was calculated to get an overall indication of the conciseness of the note. Table 4 shows the difference in mean component scores. As can be seen from the data in Table 4, the mean conciseness score, indicating whether note elements were focused and brief, increased significantly. Furthermore, the mean clearness score, indicating whether note elements were understandable to clinicians, also increased significantly.

**Table 4:** Mean component score difference between unstructured and structured documentation

**Component (number of elements for which component was used)**	**Explanation of component**	**Mean difference (95% CI)** **of mean component score**	**p-value of difference**
Sufficient information (7)	Enough information for purpose	+14.3 (10.2 – 18.4)	< 0.001*
Concise (9)	Focused and brief, not redundant	+10.7 (6.5 – 14.9)	< 0.001*
Clear (8)	Understandable to clinicians	+14.8 (10.6 – 18.9)	0.009*
Organized (3)	Properly grouped	+14.5 (7.8 – 21.2)	< 0.001*
Complete (3)	Adresses the issue	+7.9 (1.61 – 14.3)	0.014*
Ordered (1)	Order of clinical importance	+16.2 (4.5 – 27.9)	0.007*
Current (3)	Up-to-date	+24.5 (17.3 – 31.7)	< 0.001*

When analyzing the scores of the general instrument that rated the notes on a scale of one to ten, a significant increase in documentation quality was also found. Mean scores increased from 6.83 to 7.52, which was an 0.68 increase (95% CI 0.44–0.94).

## Discussion

The study offers some important insights into the impact of increased structured and standardized documentation on EHR note quality in outpatient care. In this retrospective multicenter study, our results show that structured documentation is associated with higher quality documentation. In summary, our results show a 20.0% increase measured on a 0–100 scale. Furthermore, results showed that structured notes were significantly longer than unstructured notes, but were more concise nevertheless.

This study showed an overall increase in documentation quality after the implementation of structured and standardized recording. In 8 of the 11 elements measured with the Qnote instrument, a significant increase in quality was found. This result may be explained by the fact that relevant elements and items that have to be documented are presented to the health care provider in an intuitive, uniform way. Therefore, clinicians are less likely to forget certain elements and items within the note. Furthermore, repeatedly recording in the same format ensures the physician is trained to record properly and completely. The medication element showed a minor, insignificant increase. This might be because medications were not included in notes in one center and therefore did not contribute to the observed results on this element. Additionally, minor, insignificant increases were found in physical examination and plan of care. This could be explained by the fact that the score for these elements was already high in unstructured documentation.

A recent study found variation in the quality of documentation between healthcare providers [9]. This variation could lead to inefficient documentation and the risk of patient harm from missed or misinterpreted information. Therefore, reducing this variability may also be considered relevant. The descriptive data on element scores in this study showed a trend indicating that the variation in documentation quality decreases when using structured documentation. However, some elements still showed significant variation. Therefore, implementing solutions that reduce variation in documentation quality between encounters and healthcare providers should be encouraged.

In addition, when the notes were analyzed differentiated by center, a significant increase in the quality of IOC notes was observed. This was also the case for follow-up notes in one of the two centers. This supports the conclusion that structured and standardized recording increases documentation quality, independent of a specific center or EHR vendor.

The results also show notes were longer when structured documentation was used. This could be because structured documentation contributes to including all relevant elements, or because health care providers are more reliant on CIT. CIT can be a problem if it leads to unnecessary, unorganized, or unclear information in a note and distracts the reader from the essential information buried within the note. This is known as note bloat. When considering the results of this study, there is no evidence that the longer notes were the result of note bloat. Firstly, an increase in quality in almost all elements where CIT is mainly used (problem list, past medical history, adverse reaction, social and family history) was observed. Secondly, the analysis on components used to assess the individual elements showed significant increases in clearness and conciseness. Therefore, it is safe to assume that in this study, the longer notes were not associated with note bloat and are most likely the result of more complete, and therefore higher quality, documentation.

The reports in the literature to date have mainly focused on the effect of electronic documentation versus handwritten documentation. Some studies have shown a perceived decrease in quality after implementing EHRs, identifying copy-paste functions (CPF) and note clutter as the main reasons for this quality decrease [17]. Others claim that EHRs increase note quality compared to manual recording in inpatient and outpatient care [11–13, 18]. A small number of studies have evaluated semi-structured templates that mainly use free-text documentation, comparing them to traditional templates or fully unstructured free-text notes. A small (n = 36) trial comparing outpatient notes written using a traditional template with an optimized template found mixed results, with no difference in overall quality [19]. However, the intervention notes were inferior in accuracy and usefulness, although better organized. Another study evaluating a quality improvement project to improve clinical documentation quality found no increase in quality [20]. A third, larger study did find a significant increase in inpatient documentation quality using a semi-structured template [21]. The abovementioned studies indicate that further research on this topic is warranted. However, our findings show compelling evidence that structured documentation can improve documentation quality.

This study has several strengths. This is the first study to use a validated measure instrument for outpatient notes to examine the impact of structured and standardized recording on outpatient note quality. Given the rising demand for reuse and exchange of healthcare data, structured and standardized data recording will become increasingly important. This study proves that structured documentation can also improve the quality of EHR notes. Furthermore, the increase in quality was found in two centers with different EHRs. These factors contribute to the generalizability of the results.

Another strength of this study is the method used to assess the quality of the notes. Of the instruments available in the literature that are used to assess the quality of documentation, most focus on the absence of data or only assess the global quality of the note, such as the PDSI-9 [22]. However, the Qnote instrument is based on a qualitative study in which relevant elements of an outpatient clinical note were identified [23]. Therefore, it is possible to rate the quality of all note elements independently and subsequently calculate a total score. This structured approach is likely to be more objective than other, more general rating instruments. Besides, rating elements individually benefit from being able to identify specific deficits in note quality. Because of this, improving the quality of clinical EHR notes can be conducted in a more targeted and effective way.

This study also has some limitations. Firstly, the main limitation of the retrospective nature of this study is that a causal relationship between the implementation of structured and standardized documenting cannot be established with certainty. In one center, the interval between the two study periods was several years. Therefore, the influence of other factors cannot be eliminated. In the other center, the interval between study periods is shorter, making it highly likely that implementing the standardized care pathway with structured documentation is the primary reason for the increase in note quality. Moreover, analyzing the data differentiated by center resulted in similar outcomes. Secondly, the Qnote instrument has been validated on a population of diabetic patients and not for oncological patients. However, the elements used are general and not disease- or setting-specific. Moreover, the general score given by the raters in this study showed similar or marginally lower scores than the Qnote instrument. This conclusion was also stated in the initial Qnote validation study [16]. Lastly, due to the visual similarity of structured and standardized notes, the complete blinding of study notes for raters was impossible. This might have led to an unconscious bias. However, the risk was minimized by recruiting note raters employed at another hospital.

The findings of this study support the assumption that structured documentation positively influences documentation quality. This is an important finding, given that the need for structured documentation will only increase in the near future because structured data is key in enabling the reuse of healthcare data. Data reuse will become increasingly important in health care, for various purposes, such as automated quality measurement, information exchange when referring patients to other health care centers, and less time-consuming data collection methods for scientific research. Furthermore, the use and implementation of decision support tools also require structured recording of healthcare data. The abovementioned applications of data reuse in healthcare can lead to increased efficiency and quality of healthcare. Nevertheless, there could be a concern that as data reuse becomes more important, healthcare providers are required to capture more data while providing care. This, in turn, might lead to an increased administrative burden. This should be avoided, as healthcare providers are unlikely to accept a documentation method that adds a significant burden to their workload [24]. Efforts should be made to to implement structured documentation methods within EHRs to enable data reuse while reducing the administrative burden. The results of this study raise further questions about the benefits and pitfalls of structured documentation systems, on which future studies should focus. These include the effect of the structured documentation systems on documentation time and effort, how physicians' perceptions regarding the documentation process and the EHR are influenced, and how these factors affect adoption, and how these factors affect adoption. As a result, we have started another study to answer such questions.

## Conclusion

This study demonstrated that structured and standardized recording led to an increase in the quality of notes in the EHR. Additionally, a significant increase in note length was found. Moreover, the results showed that the longer notes were also considered more clear and concise. Considering the benefits of structured data recording in terms of data reuse, it is recommended to implement structured and standardized documentation into the EHR.

## Supplementary information

Below is the link to the electronic supplementary material.

Supplementary file1 (PDF 159 KB)

## Data Availability

Data is available upon reasonable request.
