# Supplementary material for: The Impact of Structured and Standardized Documentation on Documentation Quality; a Multicenter, Retrospective Study
Source: J Med Syst. 2022 May 27;46(7):46. doi: 10.1007/s10916-022-01837-9 (PMC9135789; doi:10.1007/s10916-022-01837-9)
Supplement: Supplementary file 1 — Supplementary file1 (PDF 159 KB) [file 10916_2022_1837_MOESM1_ESM.pdf]

Unmarked = static text
Green = Imported from (or documented) elsewhere within the EHR
Yellow = documented using structured documentation form (storing structured data)
Blue = documented using structured documentation form (in free-text field)

<patient name> Patient ID: <ID>

<physician name> Progress note Visit date: <date>
<physician role>
<department>

Head and Neck Oncology intake

Present at intake:

<physician name>	Otorhinolaryngology
<physician name>	Oromaxillofacial surgery
<physician name>	Radiotherapy

Referring physician: <physician name>

Reason for visit
<free-text answer>

Imaging or other tests conducted elsewhere: <yes/no>
Conclusion(s) of imaging or other tests conducted elsewhere
<free-text answer(s)>

History

Past medical history

Diagnosis	Date
<List of all known problems>	<date of diagnosis>

Past surgical history

Surgical procedure	Laterality	Date
<List of all known surgical procedures>	<Laterality of procedure>	<Date of procecdure>

History of present illness:
Short HPI: <short free-text HPI taken by nurse practitioner>

Pain related to tumor: <yes/no>
Uses pain medication: <yes/no>
Otalgia: <yes/no>
Speech: <normal/nasal/muffled>
Hoarseness: <yes/no>
Trismus <yes/no>
Dyspnea: <yes/no>

Swelling of the neck: <yes/no>
Aspiration: <yes/no>

<Additional free-text for HPI>

Swallowing difficulties: (!) yes/no, Density of oral intake: <standardized list>
Weight: <weight in kg> / Height: <height in cm> / BMI: <BMI kg/m2> Unwanted weight loss: <yes/no>

WHO Score: <1/2/3/4>

Allergies

<list of known allergies>

Medications

Medication	Prescription	Start date
<list of all medicaments>	<prescription instructions including dose and schedule>	<start date>

Social history

Marital status: <standardized list>
Family composition: <standardized list>
Job: <standardized list>
Social support system: <free-text answer>
<additional standardized items or free-text possible>

Tobacco use	
• Smoking status:	<standardized list>
Types:	<standardized list>
Packs/day	<number>
Alcohol and drug use	
• Alcohol use:	<standardized list>
Alcohol/ per week:	<number>
Types:	<standardized list>
• Drug use:	<standardized list>
Drugs/ amount	<number>
Types:	<standardized list>

Physical examination

Extension of tumor in three planes
<free-text answer>

Oral cavity:
Dentition: <upper prosthesis/lower prosthesis/normal/sanitized/desolate/edentulous>
Left tonsil grade: <1/2/3/4/missing> Right tonsil grade: <1/2/3/4/missing>
<additional description>

Endoscopic exam:
Nasopharynx <normal/abnormal>
Base of tongue <normal/abnormal>
Adequate view of hypopharynx <yes/no>

Laryngeal mobility <normal/abnormal>
<additional description>

Examination of the neck:
Left: <none/solitary nodule/multiple nodules> <max diameter>
Right: <none/solitary nodule/multiple nodules> <max diameter>

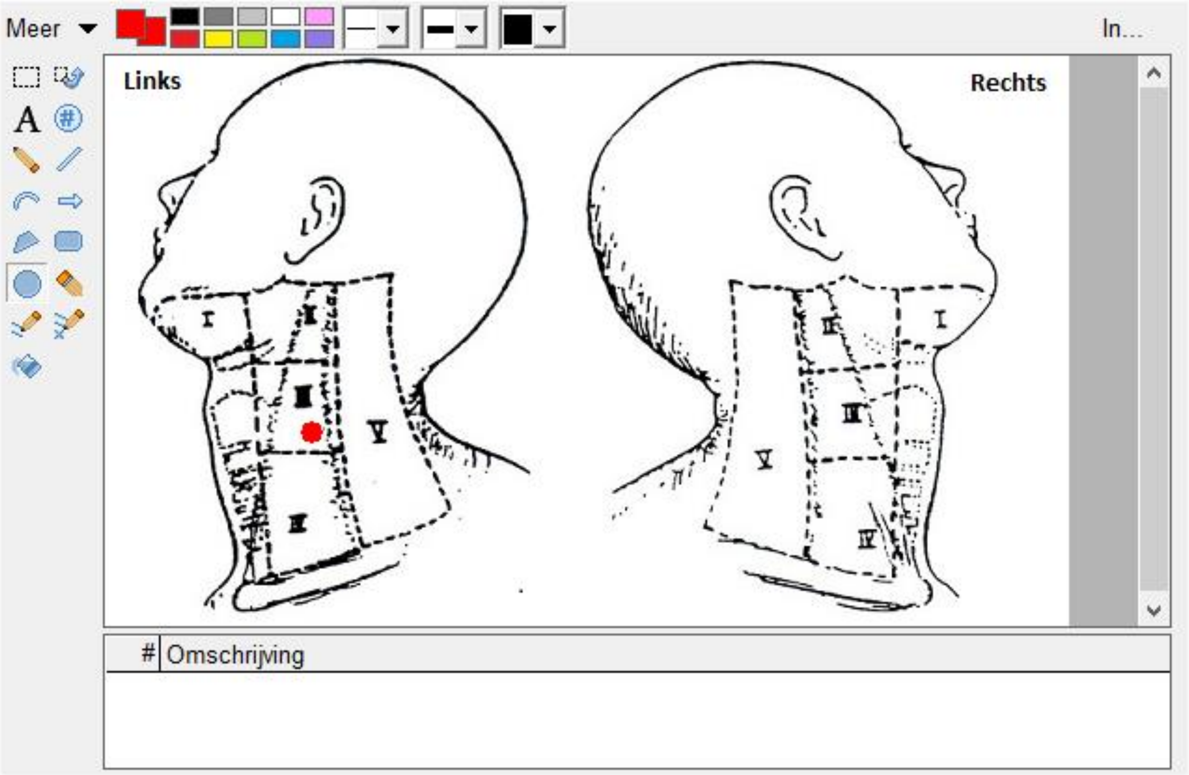

<additional description of abnormalities of neck>

Rhinoscopy
left <normal/abnormal>, right <normal/abnormal>
<additional description>

Otoscopy
left <normal/abnormal>, right <normal/abnormal>
<additional description>

Conclusion
<suspected/confirmed> <TNM-stage><tumor localization> <laterality> <primary tumor/second primary tumor/residual tumor/recurrent tumor/metastasis/benign lesion>
Preliminary treatment plan: <curative/palliative> <treatment modality 1> + <treatment modality 2 …>
<additional information>

Requested imaging, tests and appointments:
<auto-generated list of all orders placed (dependent on choices within form)>
<additional information>

Photo
<relevant images and/or photos>
